# Single-Center Retrospective Analysis of the Outcomes of Patients Undergoing Staged Peritoneal Lavage for Secondary Peritonitis

**DOI:** 10.1007/s00268-020-05455-9

**Published:** 2020-03-06

**Authors:** D. Daskalopoulou, J. Kankam, P. C. Ambe, K. Zarras

**Affiliations:** 1 Department of Visceral, Minimally Invasive and Oncologic Surgery, Marien Hospital Düsseldorf, Rochusstr. 2, 40479 Düsseldorf, Germany; 2 Department of General, Visceral and Transplantation Surgery, University of Muenster, Albert-Schweitzer Campus 1, Waldeyerstr. 1, Münster, Germany; 3 Faculty of Medicine, Witten/Herdecke University, Witten, Germany

## Abstract

**Background:**

Secondary peritonitis is associated with high rates of morbidity and mortality. Data on the effect of staged re-laparotomy or re-laparoscopy as a surgical option in the management of abdominal sepsis due to secondary peritonitis are limited and conflicting. Herein, we report the outcomes of patients undergoing staged peritoneal lavage (SPL) for secondary peritonitis in our department.

**Methods:**

This is a single-center retrospective analysis of the data of patients undergoing SPL for secondary peritonitis. SPL was performed via either re-laparotomy or re-laparoscopy. The simplified acute physiology score (SAPS II) was calculated at the time of the initial operation and for each SPL. The end points of interest included: the evolution of sepsis characterized by the SAPS II score, the mortality rate and the rate of definitive abdominal wall closure.

**Results:**

The data of 74 patients with a median age of 73 years requiring at least one SPL between 2012 and 2019 were analyzed. The median number of SPL performed was three (range 1–12). A sequential drop of SAPS II score from 41 at the initial procedure to 32 at the third SPL was documented. The overall mortality rate was 16.2%, definitive abdominal closure was achieved in all surviving patients and the median length of stay was 17.5d

**Conclusion:**

Staged re-laparotomy or re-laparoscopy with peritoneal lavage may reduce the severity of peritonitis and reduce the risk of mortality in patients with abdominal sepsis. Maintaining the abdominal wall under constant retraction using a rigid mesh while creating an open abdomen is a crucial step in achieving definite abdominal wall closure. Thus, staged peritoneal lavage may be a good surgical option for selected patients with peritonitis.

## Introduction

Secondary peritonitis (SP) is the result of the transmigration of bacteria in the abdominal cavity following the loss of integrity of the gastrointestinal tract [1]. This situation is associated with high rates of morbidity and mortality [2, 3]. Adequate surgical source control, early administration of broadband antibiotics and supportive care in a multidisciplinary setting are paramount to prevent the development of multiple organ failure, which despite aggressive management develops in up to 75% of the cases [3, 4].

Peritoneal lavage (PL), defined as irrigation of the abdominal cavity, was first introduced by Hotchkiss in 1907 aiming at diluting the content of the abdominal cavity and removing toxins [5]. Additionally, PL enables the reduction of the bacterial load and thus represents an essential aspect of surgical management of abdominal sepsis [6]. After the initial operation, staged or on demand re-laparotomy or re-laparoscopy may be indicated to manage persistent peritonitis [7]. While staged re-laparotomy (re-laparoscopy) is generally performed within 24–48 h following the initial operation, on demand re-laparotomy (re-laparoscopy) on the other hand is generally performed following clinical deterioration or lack of improvement with the need for surgical re-evaluation [7, 8].

Data on the effect of staged re-laparotomy (re-laparoscopy) as an option in the management of abdominal sepsis are limited and conflicting (7, 9–12). Staged re-laparotomy or re-laparoscopy constitutes a standard procedure for the management of abdominal sepsis in our department. Herewith, we report the outcomes of patients undergoing staged peritoneal lavage (SPL) for the management of secondary peritonitis in our department.

## Methods

This is a single-center retrospective analysis of the data of patients undergoing SPL for secondary peritonitis. The charts of the patients managed with SPL in our department within a seven years period from 2012 until 2019 were retrospectively reviewed. Patients with primary peritonitis and patients that developed tertiary peritonitis were excluded from the study.

Patients were admitted following presentation in the emergency department or following in-hospital consultations. Preoperative work-up included physical examination, blood chemistry, abdominal ultrasound sonography and computed tomography as needed. The indication to proceed to surgery was made by the attending surgeon. The means of access, that is laparoscopy versus laparotomy, was at the discretion of the attending surgeon. The decision to perform SPL was also reached by an attending surgeon. Conversion from laparoscopy to laparotomy was performed at the discretion of the senior surgeon. No difference was made between cases converted to laparotomy after diagnostic laparoscopy and cases with primary laparotomy.

As per departmental standards, all patients presenting with abdominal sepsis were put on broadband antibiotics, and the indication for intensive care management was lavishly made. Staged peritoneal lavage was performed in all cases within 24–48 h. The micro-bacterial treatment was regularly re-evaluated and adjusted based on resistance studies. Peritoneal lavage was performed with a minimum of 10 L normal saline at a temperature of 38 °C.

**Fig. 1:**
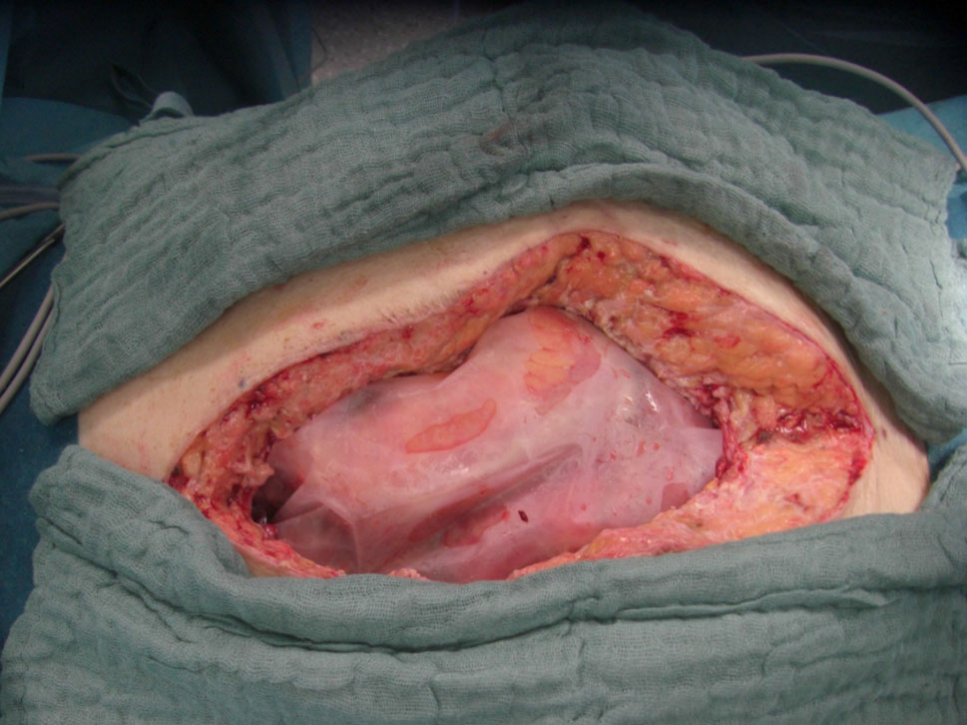
Large plastic sheet placed over the bowel

**Fig. 2:**
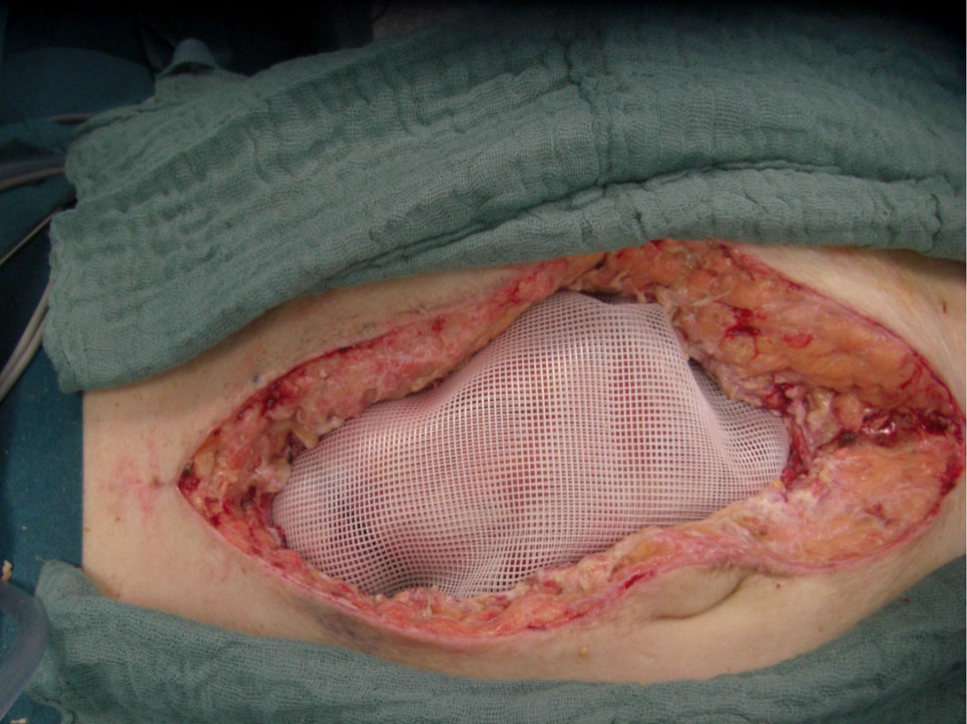
Rigid Parietex® mesh sutured onto the edges of the laparotomy wound

**Fig. 3:**
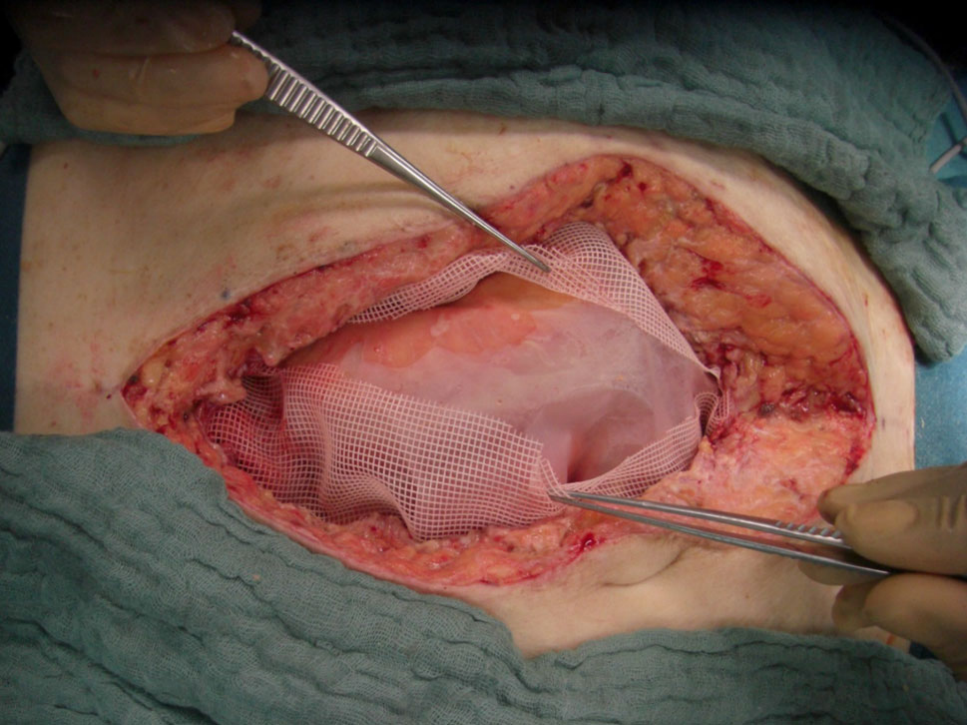
The incision in the middle of the Parietex® mesh is sutured at the end of SPL with a non-absorbable running suture

For all cases initially managed via laparoscopy, access into the abdominal cavity was gained via the initial incisions, and SPL was done in a standard fashion using an irrigation and suction device. For open cases, a large plastic sheet was placed over the bowel with lateral overlaps deep into the flanks to prevent adhesions of the bowel with the lateral abdominal wall, Fig. 1. A rigid Parietex® mesh was then sutured on the edges of the laparotomy wound to enable traction on the abdominal fascia, Fig. 2. Thus, a temporary open abdomen is created to prevent the development of abdominal compartment. This mesh was incised in the middle during re-laparotomy for SPL, Fig. 3. The incision in the middle of the Parietex® mesh was sutured at the end of SPL with a non-absorbable suture, thereby reintroducing tension on the abdominal wall. The Parietex® mesh was removed prior to definitive abdominal wall closure. Definitive closure was performed using a running slowly absorbable suture in small bite technique. The abdominal pressure was documented using the pressure in the urine bladder, which was measured and documented every 8 h. Timing of definitive abdominal wall closure was based on clinical and laboratory findings as well as improvement of peritonitis per judgment of an attending surgeon.

The evolution of sepsis was documented using the simplified acute physiology score (SAPS II) which was calculated for the initial operation and for each SPL thereafter [9–11].

Demographic features including age, body mass index (BMI), gender and clinical performance score based on the American Society of Anesthesiologists (ASA) score were collected in all cases. Clinicopathologic features including the source of the peritonitis and surgical route of access, the number of SPL, the length of hospital stay (LOS), severe complications defined as Clavien–Dindo grade III and above and the SAPS II scores in the course of the SPL were recorded.

The data generated was analyzed using the Statistical Package for the Social Sciences (SPSS® IBM version 25). All results are reported using absolute numbers, percentages, medians and ranges using a 95% confidence interval where necessary. The end points of interest included: the evolution of sepsis characterized using the SAPS II score, the mortality rate and the rate of definitive abdominal wall closure among all survivors.

## Results

The data of 74 patients (40 females and 34 males) requiring at least one SPL due to secondary peritonitis between 2012 and 2019 were analyzed. The demographic characteristics and the clinicopathologic features of the study population are summarized in Table 1.

**Table 1:** Summary of the baseline and clinicopathologic features of the study population

Features	Results
Age	
Median	73 yrs
Range	26–91 yrs
Sex	
Female	40 (54%)
Male	34 (46%)
BMI	
Median	24.9
Range	14–37
ASA	
1–2	42 (56.8%)
> 2	32 (43.2%)
Source of peritonitis	
Upper GI	29 (39.2%)
Hepatobiliary	12 (16.2%)
Lower GI	35 (47.3%)
Malignant	25 (33.7%)

*ASA* American Society of Anesthesiologist; *BMI* body mass index; *yrs* years

**Fig. 4:**
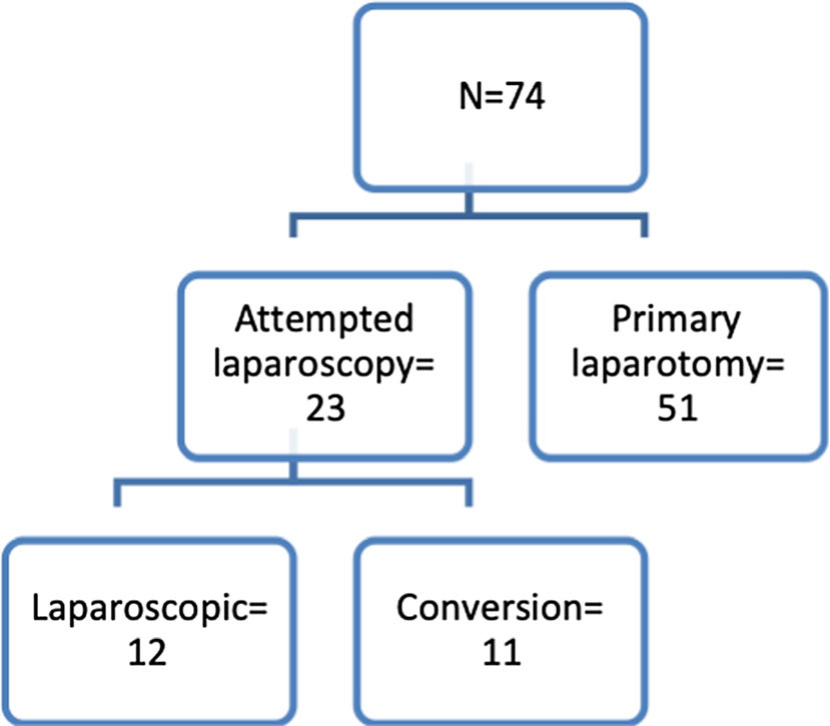
Distribution of the study population

**Fig. 5:**
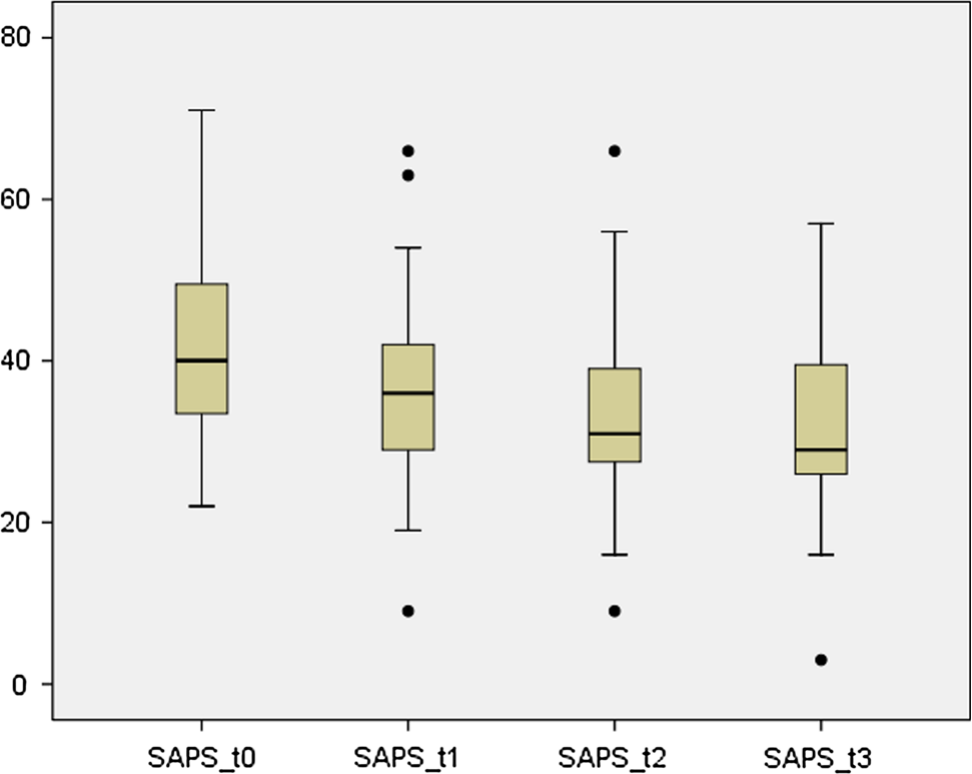
Median SAPS scores based on the median number of SPL (3)

Laparoscopy was attempted in 23 cases (31%) of which 11 were converted to open surgery, while 51 patients (68.9%) underwent primary laparotomy. Figure 4 shows the distribution of the study population. Two hundred and sixty SPL and a median of three SPL, range 1–12, were documented. The evolution of median SAP II scores in the course of SPL is presented in Fig. 5. Elevated intraabdominal pressures (> 20 mmHg) were not recorded.

Relevant complications that occurred in the course of treatment were recorded in 35 cases (47%), Table 2 [12–14]. The overall mortality rate in this study was 16.2% (12 cases). The cause of death was multiple organ failure in 10 patients and heart failure in one patient. Another patient died due necrotizing pancreatitis after completion of SPL with definitive abdominal wall closure. Definite abdominal wall closure was achieved in all surviving patients. The median LOS was 17.5 d (1–60 d).

**Table 2:** Relevant complications defined as Clavien–Dindo ≥ 3

Clavien–Dindo grade	Complication	Results
IIIA	Pleural effusion	7 (9.5%)
	Wound infection	9 (12.1%)
	Cerebral infarction	1 (1.4%)
IIIB	Postoperative bleeding	4 (5.4%)
	Abscess	3 (4%)
	Acute cholecystitis	1 (1.4%)
IVA	Acute renal failure	8 (10.8%)
	Respiratory failure	2 (2.7%)
IVB	Multiple organ failure	10 (13.5%)
V	Death	11 (14.9%)

## Discussion

The outcome of patients undergoing staged peritoneal lavage for the management of secondary peritonitis in our department was the main focus of this retrospective analysis. A sequential reduction in SAPS score was recorded in the course of SPL. SPL was associated with 16% mortality rate in this series, and abdominal wall closure was achieved in all surviving patients following SPL.

Secondary peritonitis has been shown to be associated with high rates of morbidity and mortality, and its management can be challenging [15]. Early and timely intervention is paramount to prevent multiple organ failure. Surgical source control, antibiotic therapy and supportive care are of utmost importance for the reduction of the mortality rate [16]. While the role of a multidisciplinary management is unquestionable, the need of repeated surgery remains contradictory [4, 15, 17, 18]. Current guidelines so far show no benefit of SPL over on demand re-laparotomy in the management of peritonitis [2, 17, 19–22]. Thus, SPL is not routinely performed.

In our series, SPL was performed every 24–48 h until abdominal sepsis resolved. While Holzheimer et al. explored the abdomen every 24 h [20], Teichmann et al. [23, 24] performed SPL initially every 24 h in the first week and every 48 h thereafter. In another publication by Van Ruler et al., re-laparotomy was performed every 36–48 h after the index operation [19]. Thus, our concept to perform re-laparotomy or re-laparoscopy within 24–48 h is in accordance with standard practice.

The efficacy of SPL in reducing mortality in patients with abdominal sepsis has been extensively investigated in the past. According to Koperna and Schulz [25], the mortality rate drops from 76.5 to 28% when re-laparotomy is performed within 48 h compared to after 48 h. In 1990, Wittmann et al. [26] published the results of a prospective study including 669 SPL from two institutions. An average of 6.1 procedures was reported in their study with a mortality rate of 25%. In another publication by Teichmann et al. [23], a total of 235 SPL were performed in 61 patients with peritonitis with 3.9 SPL in average and an overall mortality rate of 22.9%. In our series, 260 SPL were performed in 74 patients with a median of three SPL. The mortality rate in our series was 16% with multiple organ failure being the most common cause of death. A similar rate of mortality has been reported by Schriba et al. following SPL [21].

An intriguing finding in our study is the documented drop in sepsis (SAPS II) score in the course of SPL. A sequential drop of SAPS II score from 41 at the initial procedure to 32 at the third SPL was documented in our study. This drop in SAPS II score corresponds to a calculated drop in the risk of mortality from 26.6 to 12.8% [9–11]. Although the role of sepsis scores in the clinical decision making with regard to the management of patients with abdominal sepsis remains unclear, this finding represents an objective and measurable parameter for the efficacy of SPL in this study. Interestingly, the evolution of sepsis scores and the rate of mortality recorded in our study strongly corresponded with the estimated mortality rates based on SAPS II score.

Definitive abdominal closure was achieved in all surviving patients in this series. Definitive abdominal wall closure rates 43–73.6% have reported in a systematic review by Atema et al. This systematic review from 2015 looked at different techniques for the management of open abdomen including negative pressure wound therapy (NPWT), dynamic retention sutures, Wittmann patch, Bogota bag, mesh and zipper [27]. Low rates of definitive abdominal closure have been documented for the Bogota bag technique, mainly due to retraction of the fascial margins, while higher rates have been reported in series using NPWT with continuous suture or mesh-mediated fascial traction [28–33]. We are convinced that the amazing rate of definitive abdominal closure in our series is secondary to our technique. Suturing a rigid Parietex® mesh on to the abdominal wall maintains the abdominal wall under constant traction, thereby preventing fascial and muscular retraction while creating an open abdomen to prevent the development of abdominal compartment.

This study is mainly limited by its retrospective design and the relatively small size of the study population. Besides, all patients included for analysis were managed by a highly specialized team of surgeons according to strict institutional standards. It is not clear whether or not the results generated in this study can be readily reproduced in other institutions. More so, all patients included in this study were managed in a multidisciplinary setting. Thus, the potential effects of supportive measures including antibiotic therapy, intensive care management, and so on, in achieving these positive results cannot be evaluated. Furthermore, follow-up data are missing. Thus, data on the long-term risk of incisional hernia could not be provided. It would be very interesting to further investigate this topic in a larger population using a prospective protocol. Our study group has established a prospective database to collect data on this topic. We would be glad to share our findings in the future.

## Conclusion

Staged re-laparotomy or re-laparoscopy with peritoneal lavage may reduce the severity of peritonitis and reduce the risk of mortality in patients with abdominal sepsis. Maintaining the abdominal wall under constant retraction using a rigid mesh while creating an open abdomen is a crucial step in achieving definite abdominal wall closure. Thus, staged peritoneal lavage may be a good surgical option for selected patients with peritonitis.
